# Predicting residual cholesteatoma with the Potsic staging system still lacks evidence: a systematic review and meta-analysis

**DOI:** 10.1007/s00405-024-08478-3

**Published:** 2024-02-13

**Authors:** Klára Borbála Körmendy, Kinga Shenker-Horváth, Alexander Shulze Wenning, Péter Fehérvári, Andrea Harnos, Péter Hegyi, Zsolt Molnár, Kata Illés, Tamás Horváth

**Affiliations:** 1 Centre for Translational Medicine, Semmelweis University, Üllői út 26, Budapest, 1085 Hungary; 2 Department of Otorhinolaryngology, Head and Neck Surgery, Bajcsy-Zsilinkszky Hospital, Budapest, Hungary; 3 Department of Morphology and Physiology, Semmelweis University, Budapest, Hungary; 4 Center for Sports Nutrition Science, Hungarian University of Sports Science, Budapest, Hungary; 5 University of Veterinary Medicine Budapest, Budapest, Hungary; 6 Institute of Pancreatic Diseases, Semmelweis University, Budapest, Hungary; 7 Department of Anaesthesiology and Intensive Therapy, Semmelweis University, Budapest, Hungary; 8 Department of Anaesthesiology and Intensive Therapy, Poznan University of Medical Sciences, Poznan, Poland

**Keywords:** Congenital cholesteatoma, Potsic, Residual cholesteatoma

## Abstract

**Purpose:**

To investigate the rate of residual disease in the Potsic staging system for congenital cholesteatomas.

**Methods:**

A protocol registration was published on PROSPERO (CRD42022383932), describing residual disease as a primary outcome and hearing improvement as secondary. A systematic search was performed in four databases (PubMed, Embase, Cochrane Library, Web of Science) on December 14, 2022. Articles were included if cholesteatomas were staged according to the Potsic system and follow-up duration was documented. Risk of bias was evaluated using the Quality In Prognosis Studies (QUIPS) tool. In the statistical synthesis a random effects model was used. Between-study heterogeneity was assessed using *I*^2^.

**Results:**

Thirteen articles were found to be eligible for systematic review and seven were included in the meta-analysis section. All records were retrospective cohort studies with high risk of bias. Regarding the proportions of residual disease, analysis using the *χ*^2^ test showed no statistically significant difference between Potsic stages after a follow-up of minimum one year (stage I 0.06 (confidence interval (CI) 0.01–0.33); stage II 0.20 (CI 0.09–0.38); stage III 0.06 (CI 0.00–0.61); stage IV: 0.17 (CI 0.01–0.81)). Postoperative and preoperative hearing outcomes could not be analyzed due to varied reporting. Results on cholesteatoma location and mean age at staging were consistent with those previously published.

**Conclusion:**

No statistically significant difference was found in the proportions of residual disease between Potsic stages, thus the staging system’s applicability for outcome prediction could not be proven based on the available data. Targeted studies are needed for a higher level of evidence.

**Supplementary Information:**

The online version contains supplementary material available at 10.1007/s00405-024-08478-3.

## Introduction

The most widely used staging system for congenital cholesteatoma is the one that was described by Potsic and colleagues in 2002 [1], which is based on the extension of the disease behind an intact tympanic membrane. A cholesteatoma limited to a single tympanic membrane quadrant is referred to as stage I, while a cholesteatoma mass behind two or more quadrants without ossicular chain involvement or mastoid extension is called stage II. Stage III means that the ossicular chain is affected but without mastoid involvement, and the cholesteatoma enters the mastoid space in stage IV disease.

There are numerous advantages to adopting a staging system in cholesteatoma management. Ideally, it could allow for more precise patient counseling about disease severity and the likelihood of experiencing residual cholesteatoma, help comparing treatment results on a multi-institutional level, choose proper surgical approach, and assess the need for second-look surgery.

As congenital cholesteatoma is an uncommon disease, accounting for only 1–5% [2] of all cholesteatomas, mostly smaller case series were published in the literature, with some of them using the Potsic staging system to categorize patients [3]. As it was described when introduced, one of the dedicated goals of this staging system is outcome prediction [1]. However, the real prognostic value of this system in estimating the occurrence of residual disease remains uncertain. To obtain a higher level of evidence on the usefulness of the Potsic staging system, a joint data analysis of these studies is needed. Therefore, we aimed to assess whether the Potsic staging system was indeed indicative of the rate of residual disease and whether the extent of hearing improvement was also connected to the stage at presentation.

## Methods

The following systematic review and meta-analysis was based on recommendations of the PRISMA 2020 guideline [4] and the Cochrane Handbook [5]. The protocol of the study was created using a PICO framework [6], has been registered on PROSPERO (registration number: CRD42022383932) and was adhered to in every step.

### Inclusion and exclusion criteria

We included patients of any age and sex, diagnosed with congenital cholesteatoma (with or without symptoms) based on the Levenson criteria [7]. These criteria are the following: upon examination a pearly white mass can be observed medial to an intact tympanic membrane with a normal pars tensa and flaccida and no history of tympanic membrane perforation, no previous otologic procedures in the anamnesis, but previous occurrences of otitis media are allowed. Additional criteria for selected studies were that all patients were undergoing primary surgery, and afterwards followed up for more than 12 months, and their cholesteatoma was graded using the Potsic staging system. For the records selected for the systematic review section of our report we also included sources with a follow-up period shorter than 12 months to allow for a wider scope of publications on this rare entity that we studied.

We included published retrospective cohort studies and observational studies written in English, whereas conference abstracts, guidelines, systematic reviews of literature, meta-analyses, animal studies, case reports, case series and reports predating the publication of the Potsic staging system were excluded. Reports that included no information based on residual disease in each stage were likewise excluded, together with patients whose outcome has been individually reported but were lost to follow-up within one year of surgery.

### Information sources and search strategy

A systematic search was performed on December 14, 2022, in PubMed, Embase, Web of Science, and Cochrane Library using the following search key, without applying filters:

(“Potsic” and stag* or system or classification or congenital or pediatric) AND cholesteatoma.

Reference lists of all identified studies found eligible during text selection were reviewed for additional reports using CitationChaser [8].

### Selection process

After automatic and manual removal of duplicates using a citation manager program (EndNote 20, Clarivate Analytics, Philadelphia, PA, USA), title, abstract and full-text selection was performed by two independent review authors (BK and KSH) using the management program Rayyan [9]. After each selection step disagreements were resolved by a third reviewer (TH) and inter-rater reliability was measured with Cohen’s kappa calculation [10].

### Data collection process

The aforementioned authors (BK and KSH) independently completed the data extraction using a predesigned Excel (Microsoft Corporation, Redmond, Washington, United States) datasheet. The following data were extracted from each eligible article if available: name of the first author, year of publication, basic demographic characteristics (proportion of females, age, number of patients), number of patients assigned to each stage, the number of residual cases in these stages, surgery technique, length of follow-up period mean, median, standard deviation and range measured in months, hearing outcomes, type and location of the cholesteatoma, exclusion and inclusion criteria. Disagreements on data extraction were resolved by discussion between the authors.

### Data items

Our main outcome was the proportion of residual disease (defined as keratinizing squamous epithelium left behind in the tympanic or mastoid cavity serving as a focal point for new cholesteatoma growth) [11] in each Potsic stage. Secondary outcomes were preoperative cholesteatoma location behind the quadrants of the tympanic membrane, improvement in hearing measured by preoperative and postoperative pure tone average or air bone gap, and mean age at diagnosis. Studies were ineligible if these outcomes were either not measured or measured but not reported.

### Study risk of bias assessment

The two authors (KBK and KSH) performed the risk of bias assessment and visualized the results independently using the QUIPS tool [12]. Disagreements arising in the process were resolved through discussion.

### Effect measures and synthesis methods

To calculate the proportions, in each Potsic stage we used the total number of patients and those with residual diseases to measure effect size. We then used a random intercept meta-logistic regression model to pool proportions where the stage was a moderator variable [13]. Knapp–Hartung adjustments [14] were applied to the parameter estimates to control for the uncertainty in between-study heterogeneity. We used the maximum likelihood method to estimate the random effect heterogeneity (τ2). For the different stages where we assumed that subgroups had different τ2 values as we anticipated differences in the between-study heterogeneity in the subgroups. We assessed the difference between the subgroups with the Cochrane *Q* test [13]. *I*^2^ was used to assess the between-study heterogeneity following the recommendations of Harrer et al. [13].

### Reporting bias assessment

Funnel plots were applied to report and visualize publication bias.

### Certainty assessment

Level of evidence was ranked using the Oxford Centre of Evidence Based Medicine (OCEBM) 2011 Levels of Evidence table [15], assisted by the OCEBM Background document [16].

## Results

### Study selection

**Fig. 1:**
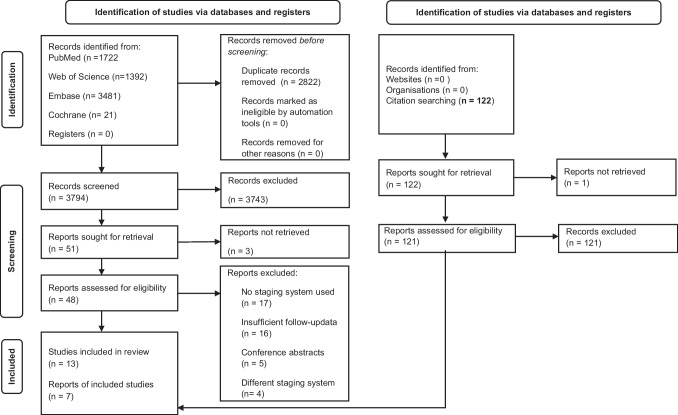
PRISMA flowchart of the selection process, which started with 6,616 articles and included 13 articles included in the review and 7 included in the meta-analysis section

Our selection process is detailed in a PRISMA flowchart pictured in Fig. 1. Inter-rater reliability according to Cohen’s kappa was 0.94 for title and abstract selection, and 1.00 for full-text selection. We have found 13 publications [17–29] that satisfied our criteria, others were excluded because they used no staging system or one different from the one proposed by Potsic et al. [1]. Reference lists of included full texts yielded no further eligible reports.

### Results and characteristics of individual studies

In our systematic review, all the included studies (their characteristics are listed in Table 1) were retrospective cohort studies, based on reviewing charts of patients previously treated for congenital cholesteatoma, diagnosed according to the criteria proposed by Levenson et al. [7].

**Table 1:** Characteristics of included studies. An overview of our key articles’ [17–29] main characteristics

	Participants (% of females, age range)	Intervention	Outcomes	Exclusion criteria (in addition to revision surgery and those listed by Levenson et al., 1986)	Follow-up time
Cho et al. 2016	93 CC (31.2% F, 1–17 yrs)	Transcanal myringotomy, tympanoplasty, CWU/CWD mastoidectomy	Age at operation, location, number of patients per year, symptoms, operative procedures, recurrence and complication rate		At least 12 months
Inokuchi et al. 2010	23 CC (48% F, 2–37 yrs)	Transmeatal tympanotomy, atticotomy and scutumplasty without mastoidectomy, CWU/CWD mastoidectomy	Location, symptoms, residual rate, hearing improvement		At least 12 months
Kim et al. 2018	12 CC involving anterior surface of the malleus and tensor tympani tendon (33% F, 2–10 yrs)	TEES, endoscope assisted procedures	Hearing, postoperative complications, residual or recurrent disease	No involvement of the anterior surface of the malleus and tensor tympani tendon, stage IV	3.5–38.0 months
Kobayashi et al. 2015	12 CC (25% F, 1–6 years)	TEES	Hearing improvement, type, size and location of cholesteatoma, complications, and recurrence	Mastoid involvement (stage IV)	3–51 months, mean 23.1 months
Lee et al. 2014	36 CC (36% F, 0.5–6 yrs)	Cholesteatoma removal with or without mastoidectomy	Age at operation, location and type of cholesteatoma, length of hospitalization residual disease, postoperative complications, mean time between initial and second-look surgery	Ossicular involvement and mastoid extension (stage III and IV)	12–56 months
Park et al. 2018	25 CC (24% F, 17 months—9 years)	TEES	Age at operation, location, size and type of cholesteatoma, hearing improvement, residual disease, postoperative complications	Mastoid involvement (stage IV)	Mean 24 months, 12.2—37.3 months
Takagi et al. 2014	71 CC (40,8% F, 1—23 years)	Transcanal or combined transcanal and transmastoid approach, planned two-stage operation, CWD mastoidectomy	symptoms, type and location of cholesteatoma, rate of residual disease		12 months to 6 years, mean period 2.1 years
Jenks et al. 2022	65 CC (29.2% F, 1.2–16 yrs)	TEES	Location, type, surgery characteristics, residual rate, hearing improvement	Stage IV	9–109.2 months
Kim et al. 2019	115 CC (37.4% F, ≤ 15 years)	Transcanal tympanoplasty, tympanomastoidectomy, tympanoplasty with atticotomy	Location, type and symptoms of cholesteatoma, age at diagnosis, residual rate		Min 24 months (mean 55.4 months)
Park et al. 2009	35 CC (22.9% F, ≤ 15 years)	Tympanoplasty, epitympanoplasty, CWU/CWD mastoidectomy, ossiculoplasty with PORP placement	CT findings, surgical findings, recurrence rate, hearing assessment		Mean 47 months, 9–128 months
Song et al. 2019	38 CC (47.4% F, 0.8–17.4 years)	Cholesteatoma removal with or without mastoidectomy	Size, type and location of cholesteatoma, rate of residual disease, hearing assessment		mean 30 months,3–88 months
Stapleton et al. 2012	82 CC (31% F, 0.3–15.3 yrs)	Transcanal tympanoplasty, endaural or postauricular tympanoplasty, CWU tympanomastoidectomy, ossicular reconstruction, second, third and fourth look surgery for 87%	Mean age at diagnosis, location, rate of residual disease and complications, hearing assessment		Mean 4.3 years, 6 months—12 years
Yamatodani et al. 2013	26 CC (42% F, ≤ 15 years)	Mastoidectomy, myringoplasty, staged tympanoplasty, ossiculoplasty	Type and location of cholesteatoma, rate of residual disease, hearing assessment		10–146 months (median 38 months)
Total	633 CC (33.6% F, 0.5–16 yrs)				

*CC* congenital cholesteatoma, *CWU* canal wall up, *CWD* canal wall down, *TEES* total endoscopic ear surgery, *PORP* partial ossicular replacement prosthesis

Due to the low incidence rate of congenital cholesteatoma, only one publication [21] included more than 100 patients. The total number of patients included in our review was 633, of whom 216 were female, their ages spanning between six months and 37 years.

A wide range of outcomes was documented apart from our primary and secondary outcomes, such as the incidence of postoperative complications, morphological findings, and the rate of open- and closed-type cholesteatoma.

### Risk of bias in studies (Supplementary material, Table 1)

As a result of assessing the risk of bias, all key articles proved to have high risk of bias according to the QUIPS tool [12]. Reasons were insufficient reporting on patients lost to follow-up, not establishing how patients were staged and whether this was done in a standardized way, and failing to define or measure potential confounders.

### Results of syntheses

The primary synthesis described in our protocol assessed whether Potsic stage at presentation was predictive of residual disease. The secondary outcome was hearing improvement and additional, not prespecified outcomes were cholesteatoma location and mean age at diagnosis.

### Results of statistical synthesis

**Fig. 2:**
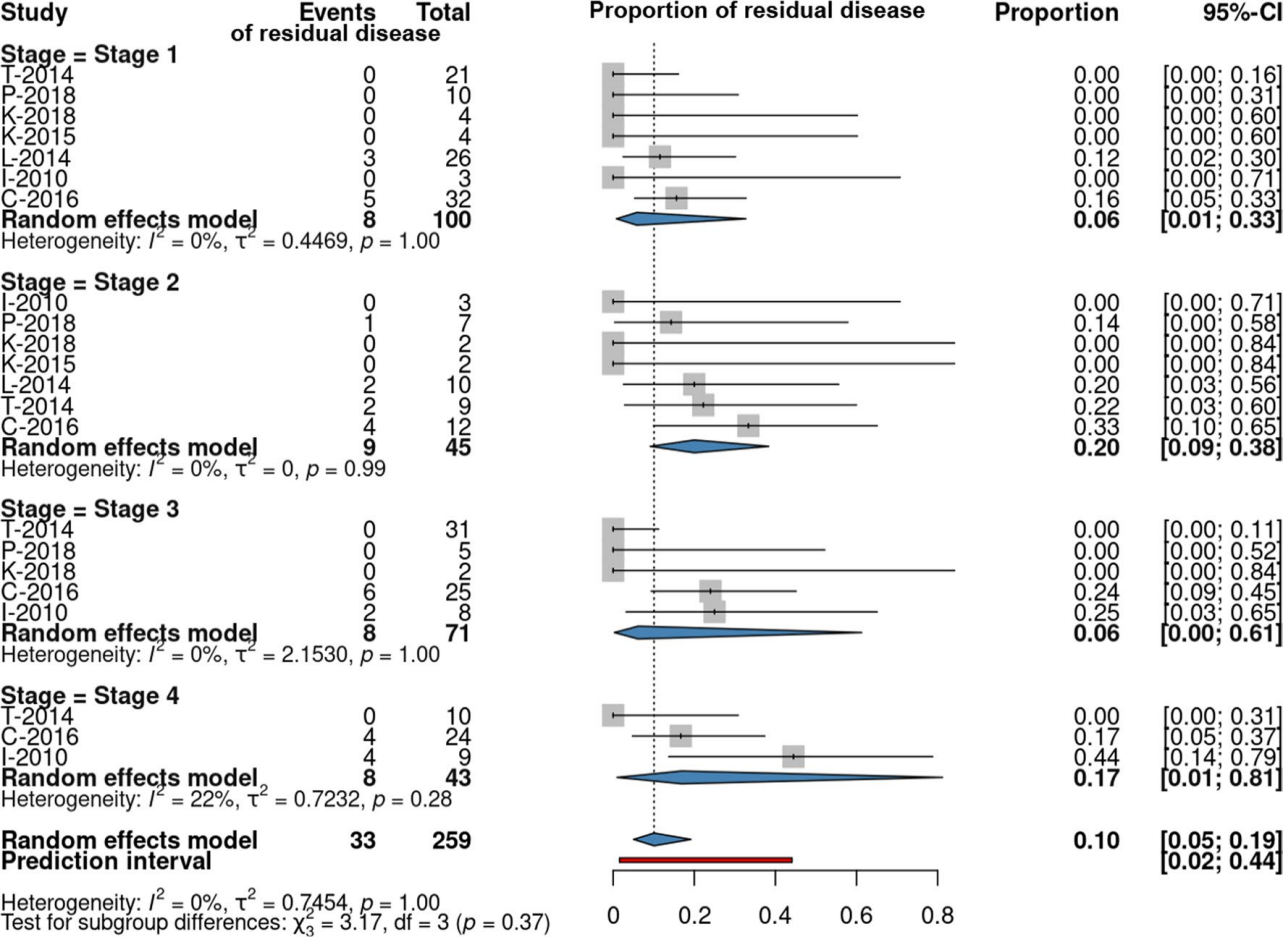
Forest plot showing the proportion of residual disease from the total number of patients after 1 year in four stages of the Potsic staging system for congenital cholesteatoma

The forest plot in Fig. 2*,* which details the results of our meta-analysis, shows the proportion of residual disease in each stage, based on data from 259 patients with a total of 33 residual cases, divided into four stages. Seven reports [17, 18, 20, 22–24, 28] were included in this analysis, where follow-up time of all patients or single patients whose individual data was published within the report exceeded 12 months after surgery.

Results of the statistical analysis showed wide confidence intervals (CI) (CI 0.01–0.33 in stage I; CI 0.09–0.38 in stage II; CI 0.00–0.61 in stage III; CI 0.01–0.81 in stage IV) and high *p* values (1.00 in stage I; 0.99 in stage II; 1.00 in stage III and 0.28 in stage IV) with *I*^2^ values (0% in stage I–III and 22% in stage IV, overall 0%), suggesting that exact heterogeneity was hard to assess. The intergroup difference was tested with *χ*^2^_3_ test (χ^2^_3_ = 3.17 *p* = 0.37). Thus, differences in the proportions of patients with residual disease in each stage were not shown to be statistically significant.

### Results of qualitative analyses

#### Location of cholesteatoma

**Fig. 3:**
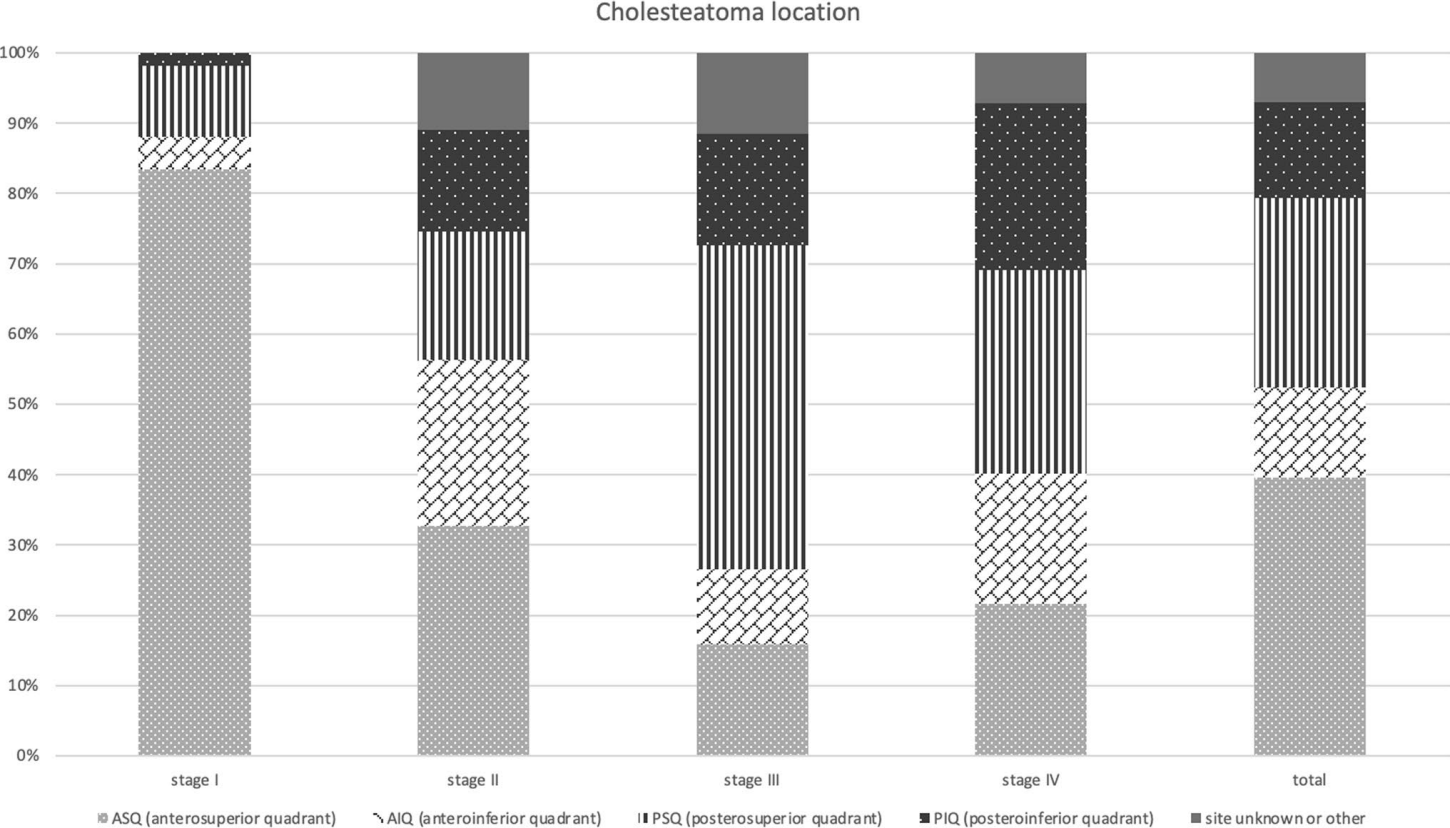
Bar chart depicting the quadrants of the tympanic membrane cholesteatomas are most commonly visible through in each stage as documented in the key articles 20, 22–26, 28, 29

Location of cholesteatoma was detailed in ten reports [20, 22–26, 28, 29]. Figure 3 shows our graph summarizing the occurrences of cholesteatoma behind each quadrant of the tympanic membrane, in the attic, and in the mastoid space. In cases where two or more locations were specified, rather than pairing them in separate groups, both were added to their significant cluster.

As a result, we have found that the anterosuperior quadrant was the most common site for cholesteatomas (214 of 563 affected quadrants), followed by the posterosuperior quadrant (163). Less common locations are the posteroinferior quadrant (45), anterior quadrants [20, 25] (not specified) (35), posterior quadrants (not specified) (29), anteroinferior quadrant (29), atticus or epitympanic space [18] (15), mastoid cavity (9), superior quadrants (not specified) (2) and central quadrant [20] (not specified) (1). In 22 cases, multiple [17, 23, 26, 29] (not specified) quadrants were reported.

#### Hearing improvement

As shown in Table 2*,* hearing results were reported in nine studies [17–25, 27–29]. All of them used a slightly different approach to assessing hearing improvement, and none of them followed the guidelines of the Committee on Hearing and Equilibrium [30]. Although analyzing hearing improvement was registered as our secondary outcome, we were unable to pool the published results.

**Table 2:** Documentation and calculation methods of hearing outcomes in our key articles [17–30]

Article (author, year)	Hearing results	Examined thresholds on audiograms (kHz)	Results reported in the articles	Note
Cho et al. 2016	Not available			
Inokuchi et al. 2010	Staged pre/postoperative PTA for 19/23 patients	0.5, 1, 2	Stage II, III, IV difference not statistically significant; Jonckheere test showed significant relationship between stage and postoperative PTA	Not reported whether results were based on bone/air conduction
Jenks et al. 2022	Staged pre/postoperative PTA, ABG for the majority of patients	0.5, 1, 2, 4	Pre- and post-operative air conduction PTA and ABG were different among Potsic I–III groups, with worse pre- and post-operative hearing results measured among Potsic stage III cases	
Kim et al. 2018	Unstaged pre/postoperative PTA (air) and PTA (bone) for 8/12 pts	0.5, 1, 2, 4		
Kim et al. 2019	Not available			
Kobayashi et al. 2015	IPD, staged pre/postoperative PTA for 4 patients	0.5, 1, 2	No ear showed any deterioration in hearing acuity, although pure-tone audiometry was performed in seven out of twelve patients	Not reported whether results were based on bone/air conduction
Lee et al. 2014	Not available			
Park et al. 2009	Staged pre/postoperative ABG for 23/35 patients	0.5, 1, 2, 3	In preoperative ABG gap a trend of the increase with advanced disease stage was detected, postoperative statistically significant improvement was not detected	
Park et al. 2018	Pre/postoperative audiogram in 1 case	0.25, 0.5, 1, 2, 4, 8		
Song et al. 2019	Preoperative PTA	Not specified		
Stapleton et al. 2012	Unstaged pre/postoperative PTA for 80/82 patients	0.5, 1, 2		
Takagi et al. 2014	Not available			
Yamatodani et al. 2013	Staged pre/postoperative AC for 14/26 patients	0.5, 1, 2, 3	A trend toward decreased rates of hearing improvement was associated with cholesteatomas of more advanced stage	
Committee on Hearing and Equilibrium Guidelines	Mean, standard deviation and range of postoperative ABG, number of decibels of closure of the ABG (both at least 1 year after treatment), the change in high-tone bone-conduction level in decibels of hearing-loss (6 or more weeks after treatment) for each case	0.5, 1, 2, 3		

*PTA* pure tone average, *ABG* air–bone gap, *IPD* individual patient data, *AC* air conduction

One study [29] showed a decreased rate of hearing improvement in more advanced stages. Similarly, in two other reports [18, 27], a significant relationship was shown between stage and postoperative pure tone average (PTA). Although no quantitative analysis was performed, results of a third publication [19] also show that pre- and post-operative hearing results were worse among stage III patients. The remaining two publications either found no significant postoperative hearing improvement [25] or showed no deterioration based on the results available for seven patients [20, 22].

#### Mean age at staging

Eight studies [17, 19, 20, 22–24, 26, 29] out of our 13 key articles had information on age at diagnosis or surgery. In three reports [19, 22, 23], age had been specified as age at first surgery. Five articles [17, 20, 22–24] had individual patient data, and three [19, 26, 29] had staged means available.

**Fig. 4:**
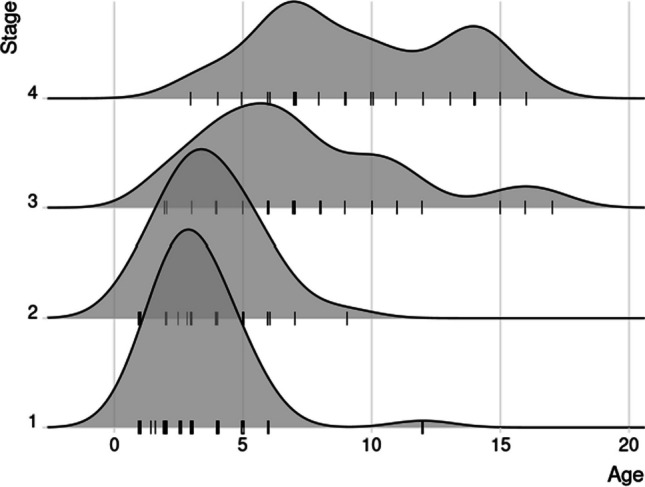
Mean age (years) at staging for patients diagnosed with congenital cholesteatoma in our key articles

To be able to calculate an approximate mean from these three articles, we have summarized (Fig. 4) the ages of patients by multiplying the mean age by the number of patients in each stage. Thus, the approximate mean age at staging came to be 3.5 years for stage I, 4.3 years for stage II, 7.5 years for stage III, and 9.4 years for stage IV based on data from 129, 53, 90, and 35 patients, respectively. The data exhibits a trend of increasing patient age as the disease becomes more extensive.

### Analysis of publication bias

Publication bias was visualized with contour enhanced funnel plots, presented in Fig. 1 of the Supplementary material. No statistical testing was conducted (as less than 10 studies were included) per the recommendations of Harrer et al. [13].

### Certainty analysis

The overall level of certainty could not be assessed according to OCEBM 2011[15] because this tool is not suitable for retrospective cohort studies, which was study design used exclusively in our key articles.

## Discussion

To summarize our results, in our meta-analysis of seven reports [17, 18, 20, 22–24, 28] involving 259 patients, we were unable to detect a statistically significant difference in the proportion of residual disease between the four Potsic stages, based on data from patients who were followed for at least 12 months after cholesteatoma removal. Hearing improvement could not be assessed due to varied reporting. Cholesteatomas were most frequently discovered behind the anterosuperior quadrant of the tympanic membrane, and there was a distinct trend of more advanced disease in older patients. Both outcomes were consistent with already existing results in the literature.

In their landmark study [1], Potsic and colleagues described some important criteria that an ideal staging system should meet: the categories should be simple and unambiguous with commensurable patient cohort sizes in each stage, and the staging system should also have good predictive value. From the perspective of our patients, the most important of these aspects is outcome prediction. The popularity of this staging system indicates that most of these expectations seem to be met. Although low incidence of this disease means that studies have usually included less than 100 patients, credible confirmation of the stage-based prediction needs much larger sample sizes.

This led us to perform a meta-analysis, collecting all the studies where the reported outcome was connected to the Potsic stage. Additional desired outcomes were to determine an ideal length of follow-up and assess the need for staged surgery and reexploration in early-stage disease. The clinical implication for a reliable staging system is to minimize time spent in the hospital for young children and reduce the number of operations performed on their ears by choosing the proper operation technique as a first procedure, thus optimizing surgical outcomes.

We have found no previous meta-analyses investigating the association between the rate of residual disease and Potsic stage, but several of our key articles [18, 19, 27] have found an association between advanced-stage cholesteatoma and an increased likelihood of residual disease. One article [26] found no association between stage and residual disease, and another [20] reported that the staging system does not reflect the differences between anteriorly and posteriorly located cholesteatoma.

Several patients from those included in our key articles [23, 24, 28] presented with residual disease after one year, which suggests that a follow-up period longer than 12 months would be desirable. However, in most articles that we found during our search process, some patients were lost to follow-up much sooner. In this analysis we had no choice but to exclude them to provide an optimal level of evidence, which could have been further enhanced, if more data on these patients had been available.

For our secondary outcome of hearing improvement, we planned to conduct a subgroup analysis to compare the stages. Although a guideline has already been published for reporting audiology test results [30], the nine articles [18–20, 22, 24–27, 29] in our analysis that did report on audiological results of all or some studies used different measurements (different frequency thresholds, ABG or PTA based on air conduction, bone conduction or both), which rendered their results unpoolable.

Location of cholesteatoma has been most frequently reported [17, 31–33] to be seen behind the anterosuperior quadrant, however Inokuchi et al. [18] found that patients in East Asia more frequently present with more extensive cholesteatomas involving multiple subregions compared to patients included in other analyses. Our report also found this quadrant to be the one most often affected.

Increase of cholesteatoma size had previously been linked to older age [34], indicating the expansive nature of cholesteatoma. In our analysis, we have also found a connection between advanced disease and older age.

We must highlight that all the included studies had a high risk of bias. As there are no dedicated tools available for reports on staging systems, we have decided to use QUIPS [12] to assess risk of bias, because this tool fit our study design best. However, there were several criteria of QUIPS that the included studies typically failed to meet. In these domains lack of data and deficient reporting resulted in a high risk of bias. Two domains (domain 2 and 4) of the QUIPS tool focuses on whether the reason for patients dropping out of follow-up was listed (together with attempts to collect information from them) and whether the exact duration of follow-up was described in the article. Domain 4 states that a clear definition should be given for the outcomes (in our case incidence of residual disease and extent of hearing improvement), and these are ought to be measured in the same way for all patients. In domain 5, possible confounding factors are an important point, such as different surgical techniques, which have been previously shown to potentially influence the likelihood of cholesteatoma recidivism [35]. In the future, including such data in reports is essential to generate high-level evidence.

### Strengths and limitations

Our analysis is the first comprehensive project to summarize the results of congenital cholesteatoma surgery after considerable follow-up (minimum 1 year) and to process them according to the most widely used staging system for this disease. It is also the first to examine the effectiveness of the staging system in predicting outcome, which, according to its authors is a vital feature of a good staging system. During our analysis, we have applied rigorous methodology and followed international guidelines for summarizing previously published data.

Limitations include the retrospective nature of the key articles, which led to heterogeneous surgical techniques, varied measurement of hearing outcomes, and inadequate data on follow-up and attrition. The rarity of congenital cholesteatoma resulted in small patient cohorts and few reports. Other limitations are pooled data in articles (e.g., for mean age at staging we can only calculate estimates) and diverse duration of follow-up periods, which prevented us from including hazard ratios into out forest plot and calculating the exact desirable follow-up duration, wherein residual disease can reasonably be expected.

### Implication for practice and research

Should more individual data documenting longer follow-up periods become available, it would be even more informative to calculate hazard ratios for each stage. However, at this time, based on the currently available data in the published literature this was not possible. In the future, more cases with carefully documented, long spanning individual follow-up data (especially in reports with small case numbers) are needed for a proper assessment. Durations of follow-up and the reason for losing patients in the process should also be documented.

In a clinical setting, second-look surgery should not be omitted purely based on stage; however, since the staging system’s abilities seem more promising for other outcomes, it is still preferable to stage cholesteatomas using this system.

## Conclusion

In our systematic review and meta-analysis, we found that although the staging system published by Potsic et al. theoretically meets the authors’ first three criteria for a good staging system, based on data published so far, we cannot confirm its prognostic value. Therefore, prospective, well-designed studies are necessary with rigorously documented follow-up to determine this staging system’s true ability to predict occurrence of residual disease.

### Supplementary Information

Below is the link to the electronic supplementary material.

Supplementary file1 Supplementary material figure 1. Contrast enhanced funnel plot to visualize publication bias. Supplementary material figure 2. Visualization of the risk of bias assessment using the QUIPS tool. (DOCX 1129 KB)

## Data Availability

The datasets used in this study can be found in the full-text articles included in the systematic review and meta-analysis.
