# Supplementary material for: Predicting residual cholesteatoma with the Potsic staging system still lacks evidence: a systematic review and meta-analysis
Source: Eur Arch Otorhinolaryngol. 2024 Feb 13;281(7):3557–68. doi: 10.1007/s00405-024-08478-3 (PMC11211107; doi:10.1007/s00405-024-08478-3)
Supplement: Supplementary file 1 — Supplementary file1 Supplementary material figure 1. Contrast enhanced funnel plot to visualize publication bias. Supplementary material figure 2. Visualization of the risk of bias assessment using the QUIPS tool. (DOCX 1129 KB) [file 405_2024_8478_MOESM1_ESM.docx]

**Predicting residual cholesteatoma with the Potsic staging system still lacks evidence: a systematic review and meta-analysis**

**Short running title:** Prediction based on Potsic staging system

**Authors:**

Klára Borbála Körmendy MD^1,2^ (orcid: 0000-0002-1534-2201), Kinga Shenker-Horváth^1,3,4^, Alexander Shulze-Wenning MD^1^, Péter Fehérvári^1,5^, Andrea Harnos^1^, Prof. Péter Hegyi MD, PhD^1,6^, Prof. Zsolt Molnár MD, PhD, EDAIC^1,7,8^, Kata Illés MD^1,2^(orcid: 0000-0002-2939-060X), Tamás Horváth MD PhD^1,2^(orcid: 0000-0001-9617-8628)

^1^Centre for Translational Medicine, Semmelweis University, Budapest, Hungary

^2^Bajcsy-Zsilinkszky Hospital, Department of Otorhinolaryngology, Head and Neck Surgery, Budapest, Hungary

^3^Department of Morphology and Physiology, Semmelweis University, Budapest, Hungary

^4^Center for Sports Nutrition Science, Hungarian University of Sports Science, Budapest, Hungary

^5^University of Veterinary Medicine Budapest, Budapest, Hungary

^6^Institute of Pancreatic Diseases, Semmelweis University, Budapest, Hungary

^7^Department of Anaesthesiology and Intensive Therapy, Semmelweis University, Budapest, Hungary

^8^Department of Anaesthesiology and Intensive Therapy, Poznan University of Medical Sciences, Poznan, Poland

**Corresponding author:**

Tamás Horváth MD, PhD

Postal address: 1106 Budapest, Maglódi út 89-91.

E-mail address: [horvath.tamas@bajcsy.hu](mailto:horvath.tamas@bajcsy.hu)

***Supplementary material figure 1.*** Contrast enhanced funnel plot to visualize publication bias


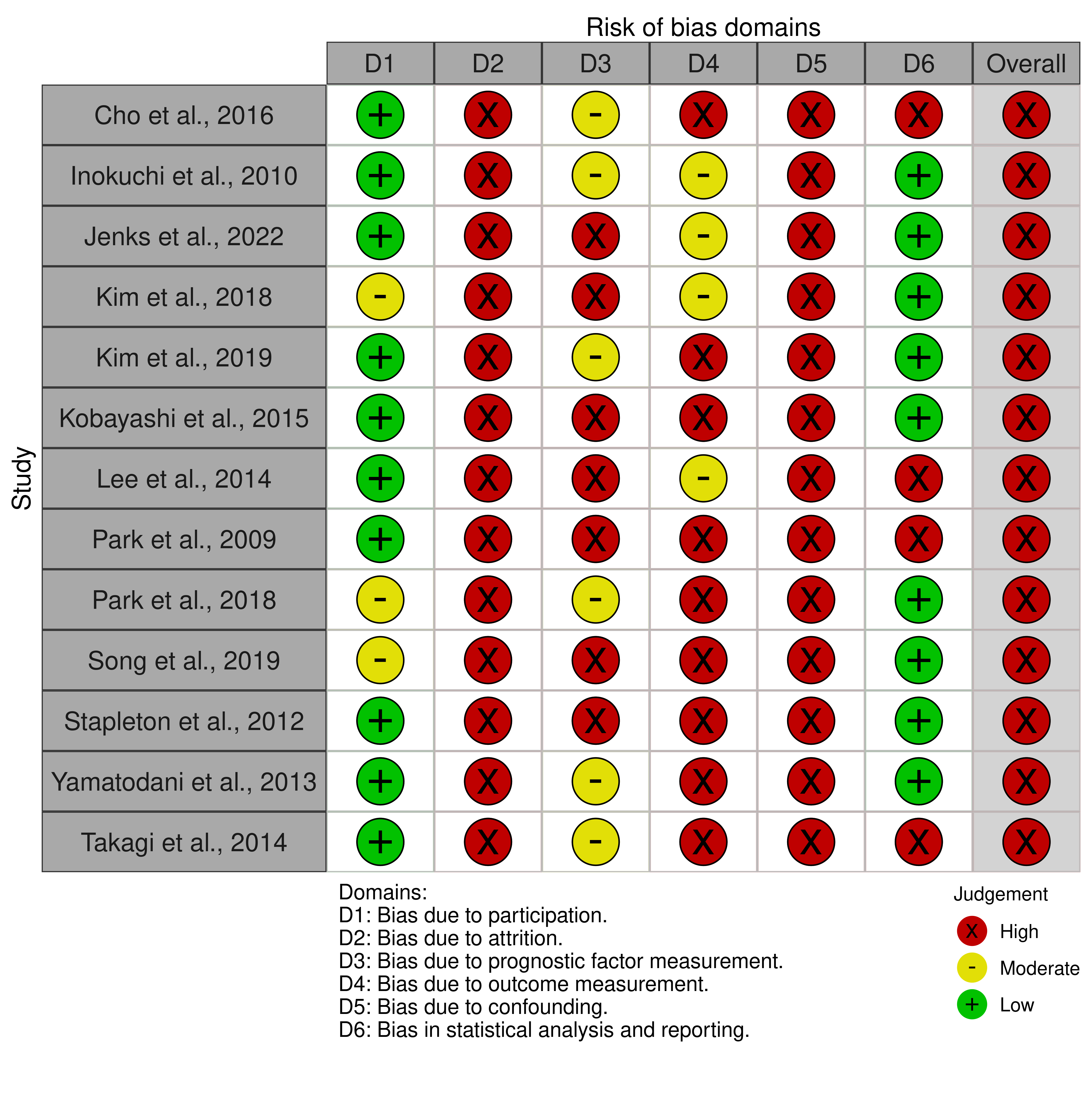


***Supplementary material figure 2.*** Visualization of the risk of bias assessment using the QUIPS tool.
